# EMF treatment delays mesenchymal stem cells senescence during long-term *in vitro* expansion by modulating autophagy

**DOI:** 10.3389/fcell.2024.1489774

**Published:** 2024-10-07

**Authors:** Wenxiang Cai, Yifan Xiao, Jiyuan Yan, Hao Peng, Chang Tu

**Affiliations:** ^1^ Department of Orthopedics, Renmin Hospital of Wuhan University, Wuhan, Hubei, China; ^2^ Department of Pathology and Pathophysiology, School of Medicine, Jianghan University, Wuhan, Hubei, China; ^3^ Hubei Key Laboratory of Cognitive and Affective Disorders, School of Medicine, Institute of Biomedical Sciences, Jianghan University, Wuhan, Hubei, China; ^4^ Department of Orthopedics, The Affiliated Hospital of Southwest Medical University, Luzhou, Sichuan, China

**Keywords:** electromagnetic fields, mesenchymal stem cells, senescence, autophagy, rejuvenation

## Abstract

**Introduction:**

Bone marrow mesenchymal stem cells (BMSCs) are widely used in tissue engineering and regenerative medicine as seed cells. Due to low amount in bone marrow, BMSCs must be expanded and cultured *in vitro* before application. However, the senescence of stem cell caused by long-term *in vitro* culture greatly limits its efficacy of transplantation.

**Methods:**

In this study, we propose an approach based on electromagnetic fields (EMF) treatment to rejuvenate aged BMSCs due to long-term *in vitro* culture. Aged BMSCs were treated with sinusoidal EMF (50 Hz, 0.4 mT), and stem cell senescence, cell proliferation, cell differentiation, cell stemness and autophagy level were detected. Additionally, aged BMSCs-laden hydrogels were transplanted into the rat critical-sized calvarial defect with or without EMF treatment. The bone formation was evaluated 8 weeks after surgery.

**Results:**

Our results indicated that the BMSCs age significantly after long-term *in vitro* passaging. The self-renew, multiple differentiation capacity, senescence phenotypes and stemness of aged BMSCs are partly reversed by EMF treatment with a frequency of 50 Hz and strength of 0.4 mT. Moreover, declined autophagy level is observed in BMSCs during long-term *in vitro* passaging and BMSCs senescence is closely associated with autophagy regulation. Additionally, the mechanistic investigation reveals that EMF treatment rejuvenate senescent BMSCs by enhancing autophagy. Furthermore, EMF treatment significantly promote the therapeutic effect of long-term passaged BMSCs on bone formation *in vivo*.

**Conclusion:**

Overall, our study identifies a practical approach for the rejuvenation of old BMSCs and may provide a promising candidate in tissue engineering and stem cell therapy.

## 1 Introduction

Due to low immunogenicity and promising potential of self-renew and differentiation, bone marrow mesenchymal stem cells (BMSCs) are extensively used in regenerative medicine (Le Blanc and Mougiakakos, 2012). Under certain conditions, BMSCs can differentiate into osteoblasts, myoblasts, tenocytes, chondrocytes, adipocytes and other mesoderm cells (Fu et al., 2019). Until now, a huge number of clinical trials based on MSCs therapy had been conducted worldwide (Maldonado et al., 2023). In most cases, tens of millions of BMSCs are required for each therapy. Considering the lack of bone marrow source and the extreme low proportion of BMSCs contained in it, *in vitro* expansion is an indispensable step for BMSCs application (Derubeis and Cancedda, 2004).

Indeed, MSCs have already initiated the aging process at the beginning of the *in vitro* culture. During the long-term *in vitro* passaging, cells display abnormal morphology, excessive cytoplasm granules and decreased telomere length (Bonab et al., 2006). In the meantime, long-term expansion contributes to the loss of MSCs self-renewal and multiple differentiation potential (Kim et al., 2009). It is reported that senescence occurred during the *in vitro* passaging greatly hinders the efficacy of BMSCs transplantation in circulatory disorders, respiratory disorders, nervous disorders, blood disorders and skeletal disorders (Shuai et al., 2016). So far, various strategies including hypoxia induction, exogenous proteins application and genetic reprogramming have been used to rejuvenate MSCs senescence during long-term *in vitro* expansion. However, owing to fluctuated environmental conditions, short half-life of exogenous molecules and potential gene mutation risk, the outcomes are still unsatisfactory (Eliasson et al., 2010; Chen et al., 2009; Fazel et al., 2008). Therefore, it is necessary for us to excavate more reliable tactics to rejuvenate MSCs during long-term *in vitro* expansion.

As a safe and noninvasive physical stimuli, electromagnetic fields (EMF) treatment proves its fundamental functions in many cellular processes (Mahaki et al., 2019). EMF with different parameters can modify the transmembrane ion channels, modulate cell cycle, regulate ROS level and activate signal pathways in stem cells (Maziarz et al., 2016). Previous study indicated that sinusoidal EMF with a frequency of 15 Hz promoted the BMSCs proliferation and osteogenic differentiation *in vitro* (Song et al., 2014). Additionally, EMF exposure preserves stemness via mediating somatic cell reprogramming to pluripotent stem cell (Baek et al., 2014). Recently, EMF was proved to have a persistent effect on BMSCs. For instance, the capacity of self-renew and differentiation of BMSCs was enhanced even long after the EMF exposure (Tu et al., 2018). These studies indicate the potential use of EMF in rejuvenating aged MSCs.

Autophagy is recognized as a highly conserved catabolic process that is essential for maintaining cell homeostasis (Levine, Mizushima, and Virgin, 2011). It is the primary intracellular degradation system through which cytoplasmic redundant components or damaged organelles are transported to the lysozyme for self-degradation (Mizushima and Komatsu, 2011). Autophagy represents as one of the hallmarks of senescence, which is related to the decline of the number and function of stem cells (Revuelta and Matheu, 2017). Reduced regenerative capacity and autophagy level are simultaneously observed in aged stem cells. When senescence stem cells were treated with autophagy activator rapamycin, the regenerative capacity of the cells was significantly enhanced (Ho et al., 2017). Besides, conditional knockout of autophagy-related gene 7 (ATG7) triggers stem cell senescence due to loss of protein homeostasis (Garcia-Prat et al., 2016). Moreover, autophagy related pathways such as mTOR/STAT3 and MAPK have been used as therapeutic targets for stem cell aging (Neves et al., 2017). These above suggest autophagy plays an important role in stem cell aging and strategies targeting autophagy may make breakthrough in rejuvenating aged MSCs due to long-term *in vitro* expansion.

Several studies revealed the connection of EMF treatment and autophagy. Low frequency EMF exposure promoted autophagy in human neuroblastoma cells via increasing the expression of autophagy related proteins including Beclin-1, LC3B and ATG7 (Marchesi et al., 2014). Additionally, 50 Hz-sinusoidal EMF triggers DNA damage-independent autophagy in hamster lung cells, thus maintaining cell homeostasis (Shen et al., 2016). Apart from these, PI3K/Akt/mTOR signal pathway can be regulated by EMF, while it is the key modulator of autophagy (Kaleagasioglu et al., 2020). However, whether EMF treatment can regulate autophagy in BMSCs remains unclear.

In this study, we expect to establish an EMF-based strategy to rejuvenate BMSCs senescence during long-term *in vitro* culture. Sinusoidal EMF (50Hz, 0.4 mT) was chose as the treatment. Comparison with untreated old BMSCs indicated EMF treatment could effectively alleviate senescent phenotypes, stemness loss and promote proliferation as well as pluripotency. The *in vivo* experiments demonstrated that EMF treatment significantly increase the therapeutic efficacy of old BMSCs. Furthermore, EMF treatment functioned via regulating autophagy. Thus, our study provides a promising way for senescent BMSCs rejuvenation which may improving MSCs therapy.

## 2 Materials and methods

### 2.1 Reagents

MSC osteogenic induction medium (OIM), adipogenic induction medium (AIM) and chondrogenic induction medium (CIM) were procured from Cyagen Bioscience. For Western blot analysis, antibodies against ATG5, ATG12, Beclin1, LC3A/B, SOX2, P-PI3K, RUNX2 were obtained from Cell Signaling Technology (Beverly, MA, United States). Antibodies specific for P62, P53, AKT, P-AKT, PI3K, mTOR, P-mTOR, GAPDH were supplied from Proteintech (Wuhan, China). Antibodies against NANOG, OCT4, ACAN, COL2, AIPOQ, PPARγ2 were purchased from Abcam (Cambridge, United States). Antibodies specific for P16 and P21 were obtained from Finetest (Wuhan, China). Antibodies specific for ALP and OCN were procured from Affinity (Cincinnati, United States). For flow cytometry analysis, the antibodies against CD34, CD44, CD45, CD29, CD90, CD105 were obtained from Elabscience Biotechnology (Wuhan, China). For immunofluorescence staining, antibodies specific for P16 and P53 were purchased from Proteintech. Antibody against P21 were obtained from ABclonal (Wuhan, China).

### 2.2 Cell culture and identification

Isolation of BMSCs was performed according to previous study (Tu et al., 2020). Briefly, whole bone marrow was collected from femurs and tibias of male Sprague-Dawley rats (6–8 weeks old). The bone marrow was suspended using growth medium (GM) which is DMEM/F12 medium (HyClone, USA) containing 10% FBS (Gibco, USA), 1% penicillin and streptomycin (Sigma-Aldrich, USA). The medium was changed every 2 days, and adherent cells were kept. Cells were passaged using 0.25% trypsin-EDTA solution (Gibco, USA) When they reached approximately 80% confluence. The fourth or thirteenth passage was used for following experiments.

For BMSCs identification, specific cell surface markers and BMSCs-multipotent potential for osteogenic, adipogenic and chondrogenic differentiation were detected.

### 2.3 EMF exposure

**FIGURE 1:**
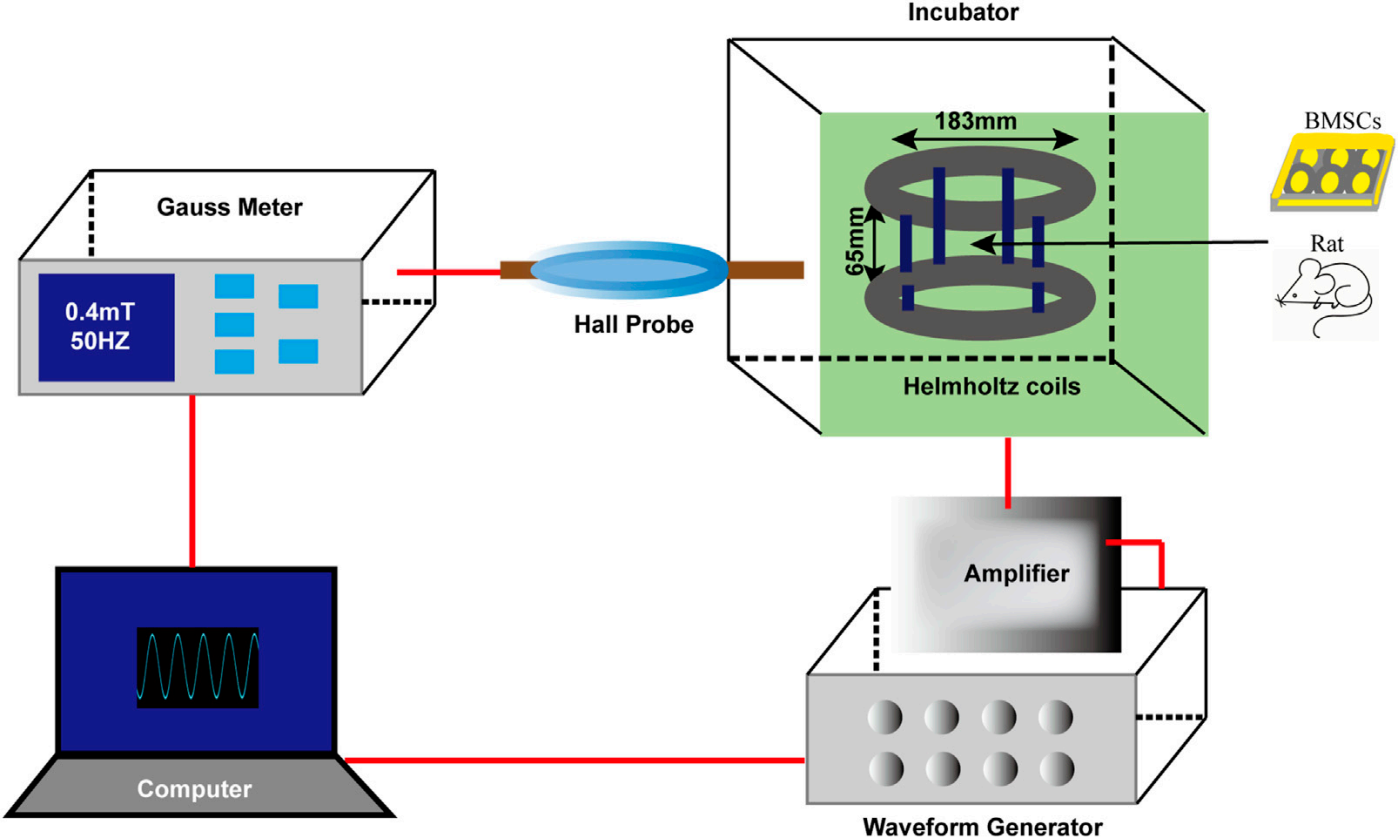
Schematic diagram of EMF device. The EMF facility contains six main parts: waveform generator, gauss meter, amplifier, hall probe, Helmholtz coils and a control compute. Sinusoidal EMF with a parameter of 0.4 mT, 50 Hz was verified by the compute and created by the waveform generator. Helmholtz coils producing uniform EMF were fixed in an incubator (37°C, 5% CO_2_). During EMF exposure, cultured rat BMSCs or experimental animals were placed in the center of the coils.

The EMF device (Figure 1) was designed and manufactured by the CH-Hall electronic devices corporation (Beijing, China). Concisely, the device comprises a waveform generator, a gauss meter, an amplifier, a hall probe, Helmholtz coils and a control computer. After finishing the parameters setting on the computer, the signals were created by the waveform generator, being amplified and transferred to the coils. The real-time frequency as well as the strength of the EMF could be measured by the gauss meter and hall probe. The coils generating EMF were placed in an incubator with a humidified atmosphere (37°C, 5% CO_2_). In this study, we used sinusoidal EMF with a parameter of 0.4 mT, 50 Hz. During exposure, cultured BMSCs or rats were placed in the center of the coils.

### 2.4 Cell proliferation assay

BMSCs at a density of 1,000 cells/well were seeded in 96-well plates. The cell proliferation was analyzed using a CCK-8 kit following the standard protocol (Boster, Wuhan, China). Concisely, 10 μL CCK-8 solution mixed with 100 μL GM was added to each well. After 2 h incubation, a microplate reader (Bio-TEK Instruments, United States) was used to measure the OD value per well at 450 nm. CCK8 assays were conducted from day o to day 6. All OD values were normalized to those of day 0.

### 2.5 Fibroblastic colony-forming assay

BMSCs at a density of 400 cells/dish were seeded in a 3.5-cm dish. The cells were cultured in GM which was half exchanged every 3 days. After 7 days of culture, cells were washed with PBS and fixed using 4% paraformaldehyde (Sigma-Aldrich, United States). Subsequently, cells were stained using 0.1% toluidine blue solution (Sigma-Aldrich, United States). Colonies including more than 50 cells were counted and the colony-forming ratio was acquired by colony numbers/400 (%).

### 2.6 EdU labeling

BMSCs at a density of 2,000 cells/well were seeded in 96-well plates. The cells were labeled with EdU solution (Beyotime, Shanghai, China) and continued culturing for 6 h. Then, cells were fixed using 4% paraformaldehyde and treated with 0.3% Triton X-100 for 15 min. Afterwards, the cells in each well were added with click reaction mixture and incubated in the dark for half an hour. The nucleus was dyed by DAPI for 10 min and cells were observed using a fluorescence microscope.

### 2.7 Alizarin Red S staining

BMSCs at a density of 5,000 cells/well were seeded in 24-well plates. The cells were cultured in GM before they reached approximately 70% confluence. Then the cells were cultured with osteogenic induction medium (Cyagen Bioscience, United States) for next 2 weeks. Afterwards, cells were washed with PBS and fixed using 4% paraformaldehyde. Finally, the induced cells were stained using 40 mM Alizarin Red S (Sigma-Aldrich).

### 2.8 Oil Red O staining

BMSCs at a density of 5,000 cells/well were seeded in 24-well plates. The cells were cultured in GM before they reached approximately 100% confluence. Then the cells were cultured with adipogenic induction medium (Cyagen Bioscience, United States) for next 2 weeks. Afterwards, cells were washed with PBS and fixed using 4% paraformaldehyde. Finally, the induced cells were stained using filtered Oil Red O (Sigma-Aldrich) at a concentration of 60%.

### 2.9 Alcian Blue staining

BMSCs at a density of 5,000 cells/well were seeded in 24-well plates. The cells were cultured in GM before they reached approximately 100% confluence. Then the cells were cultured with chondrogenic induction medium (Cyagen Bioscience, United States) for next 2 weeks. Afterwards, cells were washed with PBS and fixed using 4% paraformaldehyde. Finally, the induced cells were stained using Alcian Blue solution (pH 2.5, Sigma-Aldrich).

### 2.10 Western blot

Cells were harvested and lysed using RIPA buffer (Boster, Wuhan). Then 25 μg protein samples were transferred using PVDF membrane (Millipore, United States) with a Bio-rad blotting system. The membranes were blocked using 5% bovine serum albumin (Sigma-Aldrich) and incubated with corresponding primary and secondary antibodies. All bands were visualized using Western ECL Substrate Kit (Thermo, United States) with a Bio-rad image capture system. For signal pathways detection, cells were first serum-starved overnight and treated with or without EMF for 30 min.

### 2.11 Senescence-associated β-galactosidase (SA-β-gal) staining

To determine the senescence of BMSCs, SA-β-gal staining was employed. Briefly, BMSCs at a density of 5 × 10^4^ cells/well were seeded in 6-well plates. The cells were washed with PBS and fixed using 4% paraformaldehyde. Then cells were stained using SA-β-gal staining solution (Beyotime) according to the manufacturer’s protocol. Aged BMSCs stained in blue were photographed with an inverted microscope.

### 2.12 Immunofluorescence staining

BMSCs at a density of 5,000 cells/well were seeded in 24-well plates. Cells were washed with PBS and fixed using 4% paraformaldehyde at room temperature. Then cells were permeabilized using 0.2% Triton X-100 (Sigma-Aldrich) and blocked using 5% bovine serum albumin (Sigma-Aldrich). Next, BMSCs were incubated with primary antibodies overnight at 4°C. Afterwards, cells were incubated with appropriate fluorescent secondary antibodies in the dark. Finally, the cell nuclei were stained using DAPI for 10 min. After rinsing, cells were observed using a fluorescence microscope.

### 2.13 Plasmid construction and transfection

The RNA sequences of ATG5 siRNAs were designed and synthesized by HanBio (Shanghai, China). Briefly, BMSCs were cultured in a 6-well plate (2 × 10^5^ cells per well). Lipofectamine 2000 (Invitrogen) was used to deliver siRNA into BMSCs following the manufacturer’s instructions. 24 h post transfection, the inhibitory efficiency of specific silencing was examined by the Western blot and qPCR. For the experiment with ATG5 overexpression adenovirus infection, the BMSCs were infected with adenovirus (HanBio) at a multiplicity of infection (MOI) of 60 for 4 h and the efficiency of ATG5 overexpression was observed 24 h post infection. Overexpression was also assessed using Western blot analysis and qPCR.

### 2.14 Rat critical-sized calvarial defect model

All animal experiments were in accordance with Wuhan University Committee on the Use and Care of Animals. The operation procedure was carried out as described before (Dubus et al., 2022). Briefly, male SD rats (weigh 280–320 g) were anesthetized through intraperitoneal injection of 3% pentobarbital. A 1 cm sagittal incision down to the periosteum was made on the skull and a 6-mm diameter (critical-size) defect was created on single side of the parietal bone using a micro-trephine. The 10% gelatin methacryloyl (GelMA) hydrogels (Sigma-Aldrich) was used as a carrier of BMSCs. All uniform defects were randomly allocated into four groups: 1) Control group including acellular GelMA hydrogel; 2) P4 group including GelMA hydrogel seeded with BMSCs at passage 4; 3) P13 group including GelMA hydrogel seeded with BMSCs at passage 13; 4) P13+EMF group including P13 BMSCs-laden GelMA hydrogel combined with EMF treatment (50 Hz, 0.4 mT, 4 h/day). All rats fully recovered 24 h after surgery. Efforts were made to minimize animal sufferings.

### 2.15 Micro-CT scanning

Six rats per group were sacrificed by injection of an overdose of anesthetics at 8 weeks after surgery. The harvested rat craniums were fixed using 4% paraformaldehyde for 3 days. Then the fixed samples were scanned using a micro-CT (μCT 40, Scanco 274 Medical, Switzerland). The defect region as well as the surrounding bone was reconstructed using VGStudio software at a constant threshold (a voltage of 80 kV, a current of 145 mA, and a resolution of 9 µm pixel^−1^). The bone volume/total volume (BV/TV) as well as the bone mineral density (BMD) within the bone defect of each group were calculated and analyzed.

### 2.16 Histological evaluation

Following CT scanning, all specimens were decalcified using 10% EDTA solution for 1 month. Then samples were dehydrated in graded ethanols, embedded within paraffin and cut into 5-μm slices. The slices were stained with HE as well as Masson’s trichrome. New bone area fraction was calculated as new bone area/defect area within the defect of each section. Immunohistological staining was also performed using antibodies against ALP.

### 2.17 Statistical analysis

Data were presented as mean ± SD. Statistical analysis were evaluated by Student’s t test or one-way ANOVA followed by Tukey *post hoc* test. For quantitative analysis of Western blot, Immunofluorescence staining and Immunohistological staining, ImageJ software was employed. *p* < 0.05 was recognized as statistically significant.

## 3 Results

### 3.1 EMF preserved the self-renewal capacity and stemness of BMSCs during long-term *in vitro* passaging

Harvested primary BMSCs were firstly identified via detecting cell surface markers and multipotent potential for differentiation. As shown in Supplementary Figure S1, primary cells were positive for CD29, CD44, CD90, CD105 while negative for CD34 and CD45. Meanwhile, under specific induction medium, cells were able to differentiate to the osteogenic, adipogenic and chondrogenic lineages.

**FIGURE 2:**
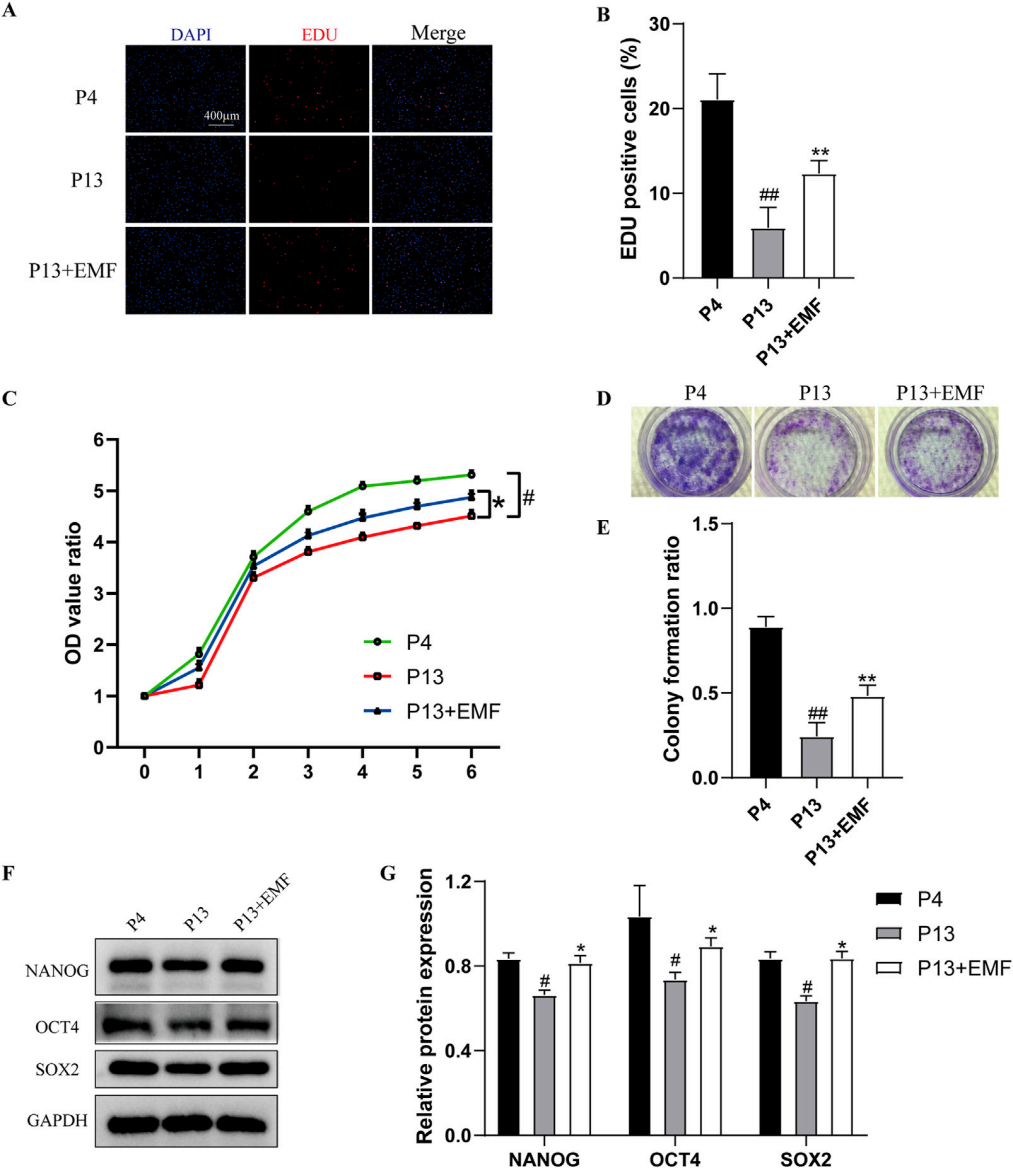
EMF exposure prevents the decreased proliferation and stemness loss of long-term passaged BMSCs **(A)** EdU staining and **(B)** percentage of EdU-positve cells of P4, P13 and 24 h EMF-stimulated P13 BMSCs. (n = 3). **(C)** Proliferation of P4, P13 and EMF-stimulated P13 BMSCs were detected from day o to day 6 (n = 3). **(D)** Toluidine blue staining and **(E)** colony formation ratios of P4, P13 and EMF-stimulated P13 BMSCs after 7-day culture (n = 3). **(F)** Western blot and **(G)** Quantitative analysis of pluripotency proteins including NANOG, OCT4, SOX2 in P4, P13 and 24 h EMF-stimulated P13 BMSCs. GAPDH was used as the internal controls (n = 3). ^#^ *p* < 0.05 and ^##^ *p* < 0.01 vs. P4 group; *p < 0.05 and **p < 0.01 vs. P13 group.

To determine the effects of EMF on proliferation capacity of BMSCs during long-term *in vitro* expansion, we chose rat BMSCs cultured for four passage (P4) and 13 passages (P13) for analysis. According to the EdU incorporation analysis, lower percentage of proliferating cells was observed in P13 BMSCs compared with P4 BMSCs, while 24 h EMF treatment could improve the decreased proliferation of P13 BMSCs (Figures 2A, B). We also performed the CCK-8 (Figure 2C) and fibroblastic colony-forming (Figures 2D, E) assay, BMSCs showed decreased proliferation rate as well as lower formation of colony forming units during the long-term *in vitro* culture. However, EMF could partly reverse this change. NANOG, OCT4 and SOX2 are three key transcription factors for maintaining cell stemness. From the Western blot analysis, P13 BMSCs exhibited reduced expression of NANOG, OCT4 and SOX2 compared with P4 BMSCs. Interestingly, 24 h EMF exposure alleviated the stemness loss of long-passaged BMSCs (Figures 2F, G).

### 3.2 EMF preserved the differentiation potential of BMSCs during long-term *in vitro* passaging

**FIGURE 3:**
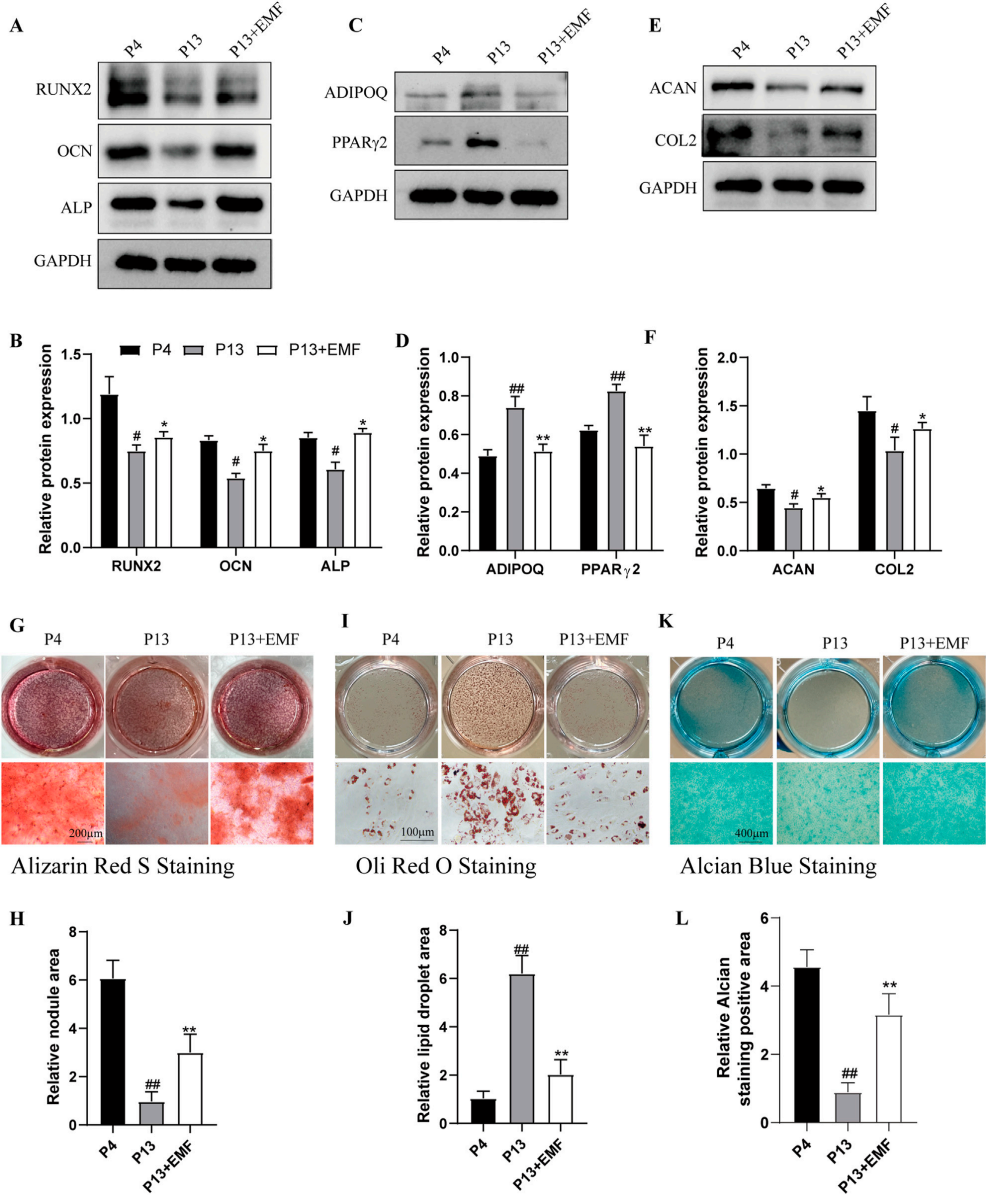
EMF exposure prevents the decreased multiple differentiation potential of long-term passaged BMSCs. BMSCs of three groups were cultured in inductive medium for 7 days. **(A)** Western blot and **(B)** Quantitative analysis of osteogenesis associated proteins including RUNX2, OCN, ALP in P4, P13 and 7-day EMF-stimulated P13 BMSCs. GAPDH served as the loading control (n = 3). **(C)** Western blot and **(D)** Quantitative analysis of adipogenesis associated proteins including ADIPOQ, PPARγ2 in P4, P13 and 7-day EMF-stimulated P13 BMSCs. GAPDH served as the loading control (n = 3). **(E)** Western blot and **(F)** Quantitative analysis of chondrogenesis associated proteins including ACAN, COL2 in P4, P13 and 7-day EMF-stimulated P13 BMSCs. GAPDH served as the loading control (n = 3). BMSCs of three groups were cultured in inductive medium for 14 days. **(G)** Alizarin Red S staining and **(H)** Quantitative analysis of mineralized nodule area among three groups (n = 3). **(I)** Oil Red O staining and **(J)** Quantitative analysis of lipid droplet area among three groups (n = 3). **(K)** Alcian Blue staining and **(L)** Quantitative analysis of staining positive area among three groups (n = 3). ^#^ *p* < 0.05 and ^##^ *p* < 0.01 vs. P4 group; *p < 0.05 and **p < 0.01 vs. P13 group.

We then explored the effects of EMF on differentiation potential of BMSCs during long-term *in vitro* culture. P4 BMSCs, P13 BMSCs with or without EMF exposure were cultured in osteogenic induction medium, adipogenic induction medium and chondrogenic induction medium respectively. After 7 days culture in inductive medium, Western blot analysis indicated that P13 BMSCs exhibited lower expression of RUNX2, OCN, ALP (Figures 3A, B), ACAN, COL2 (Figures 3E, F) and higher expression of ADIPOQ and PPARγ2 (Figures 3C, D) compared with P4 BMSCs. However, EMF treatment could significantly partly reverse these changes. After 14 days culture in inductive medium, we next performed the Alizarin Red staining (Figures 3G, H), Oil Red O (Figures 3I, J) staining and Alcian Blue staining (Figures 3K, L) in three groups, results showed the same tendency. Taken together, EMF exposure effectively rescued the decline of differentiation potential of BMSCs during long-term *in vitro* expansion.

### 3.3 EMF inhibited the BMSC cell senescence after long-term *in vitro* passing

**FIGURE 4:**
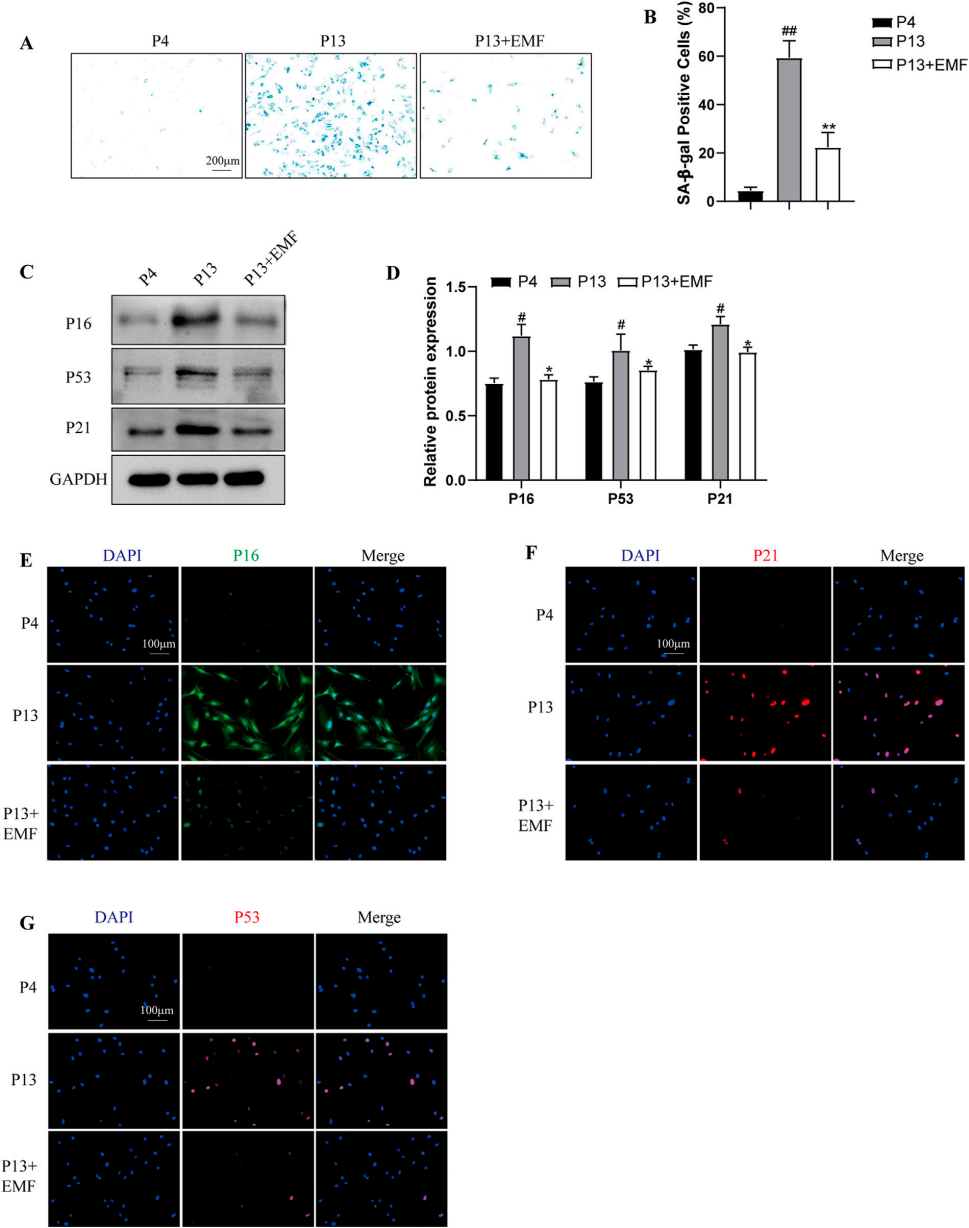
EMF exposure prevents the cell senescence of long-term passaged BMSCs **(A)** SA-β-gal staining and **(B)** percentage of SA-β-gal-positive cells of P4, P13 and 24 h EMF-stimulated P13 BMSCs. (n = 3). **(C)** Western blot and **(D)** Quantitative analysis of senescence makers including P16, P53, P21 in P4, P13 and 24 h EMF-stimulated P13 BMSCs. GAPDH was employed as the loading control (n = 3). **(E)** P16, **(F)** P21 and **(G)** P53 were further observed by Immunofluorescence among three groups. ^#^ *p* < 0.05 and ^##^ *p* < 0.01 vs. P4 group; *p < 0.05 and **p < 0.01 vs. P13 group.

To verify the effects of EMF exposure on BMSCs cell senescence, we firstly conducted the SA-β-gal staining. Results indicated P13 BMSCs showed notably higher SA-β-galactosidase activity compared with P4 BMSCs, while 24 h EMF treatment could significantly ameliorate this process (Figures 4A, B). Furthermore, By Western blot analysis, senescent BMSCs (P13 BMSCs) showed lower expression of P16, P53 and P21 after 24 h EMF exposure (Figures 4C, D). We also performed the immunofluorescence staining of P16, P21 and P53 in three groups, results exhibited the same trend (Figures 4E–G).

### 3.4 Autophagy acts as an important role in cell aging during long-term *in vitro* passaging

**FIGURE 5:**
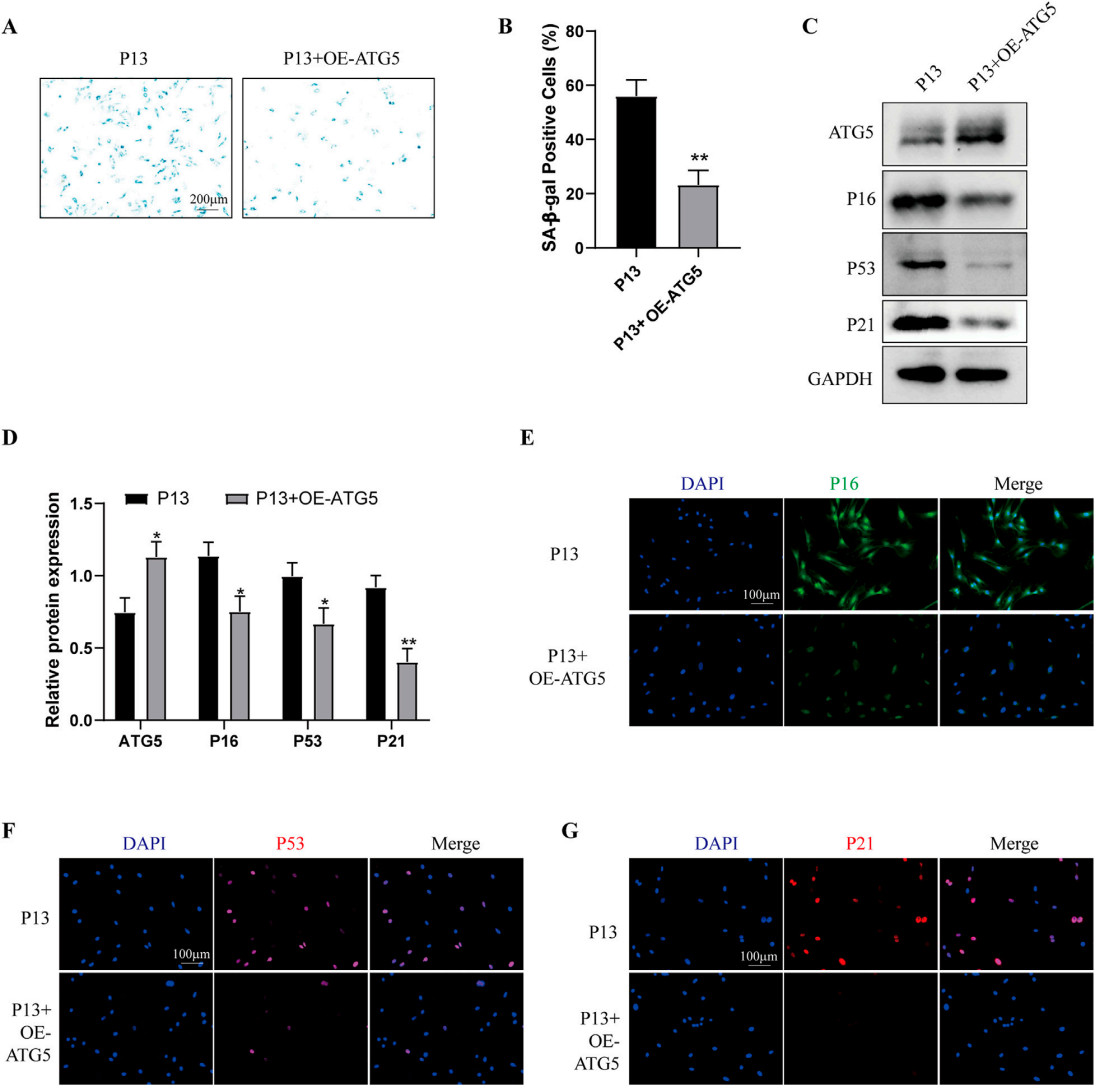
Over-expression of ATG5 alleviates the cell senescence of long-term passaged BMSCs P13 BMSCs were transfected with empty vectors (P13 group) or Ad-ATG5 adenoviruses vectors (P13 + OE-ATG5 group) for 24 h. **(A)** SA-β-gal staining and **(B)** percentage of SA-β-gal-positive cells among wo groups. (n = 3). **(C)** Western blot and **(D)** Quantitative analysis of ATG5, P16, P53, P21 among two groups. GAPDH was used as the loading control (n = 3). **(E)** P16, **(F)** P53 and **(G)** P21 were further observed by Immunofluorescence among two groups. *p < 0.05 and **p < 0.01 vs. P13 group.

**FIGURE 6:**
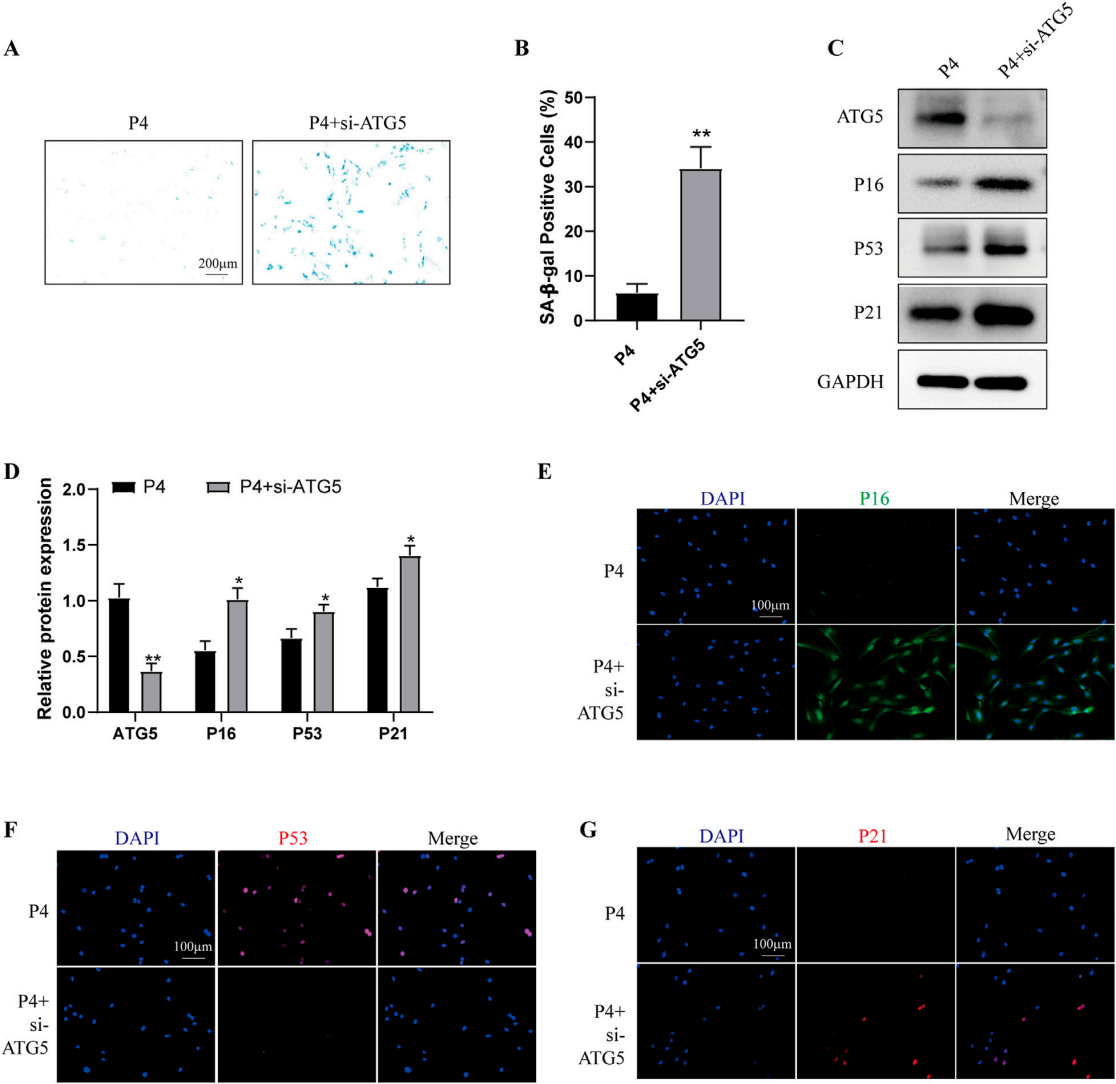
Knockdown of ATG5 aggravates the cell senescence of young BMSCs P4 BMSCs were transfected with negative control siRNA (P4 group) or ATG5 siRNA (P4 + siATG5 group) for 24 h. **(A)** SA-β-gal staining and **(B)** percentage of SA-β-gal-positive cells among wo groups. (n = 3). **(C)** Western blot and **(D)** Quantitative analysis of ATG5, P16, P53, P21 among two groups. GAPDH was used as the loading control (n = 3). **(E)** P16, **(F)** P53 and **(G)** P21 were further observed by Immunofluorescence among two groups. *p < 0.05 and **p < 0.01 vs. P4 group.

To determine the role of autophagy in cell senescence during long-term *in vitro* passaging, we firstly examined the autophagy level in P4 and P13 BMSCs. As shown in Supplementary Figures S2A, B, P13 BMSCs exhibited lower expression of ATG5, ATG12, Beclin-1, LC3B/A and higher expression of P62. Moreover, increased levels of P-PI3K, P-AKT and P-mTOR were observed in P4 BMSCs compared with those of P13 BMSCs (Supplementary Figures S2C, D). Above results suggested long-term *in vitro* expansion of BMSCs was accompanied with reduced autophagy. We then used autophagy inducer rapamycin in P13 BMSCs, cells showed decreased SA-β-galactosidase activity after rapamycin treatment (Supplementary Figure S3A, B). Meanwhile, the Western blot and immunofluorescence staining detection suggested aging-associated markers including P16, P53 and P21 were significantly decreased in P13 BMSCs after rapamycin stimulation (Supplementary Figures S3C–G). Additionally, over-expression of ATG5 in P13 BMSCs could inhibit the SA-β-galactosidase activity (Figures 5A, B) and the level of P16, P53 and P21 (Figures 5C–G). We also examined the cell senescence in P4 BMSCs by silencing ATG5. Higher levels of SA-β-galactosidase activity (Figures 6A, B) as well as increased expression of P16, P53 and P21(Figures 6C–G) were observed in P4 BMSCs after ATG5 silencing. Taken together, our results suggested impaired autophagy contributed to the BMSCs aging during the long-term *in vitro* expansion.

### 3.5 EMF delayed BMSC cell senescence partly via promoting autophagy

**FIGURE 7:**
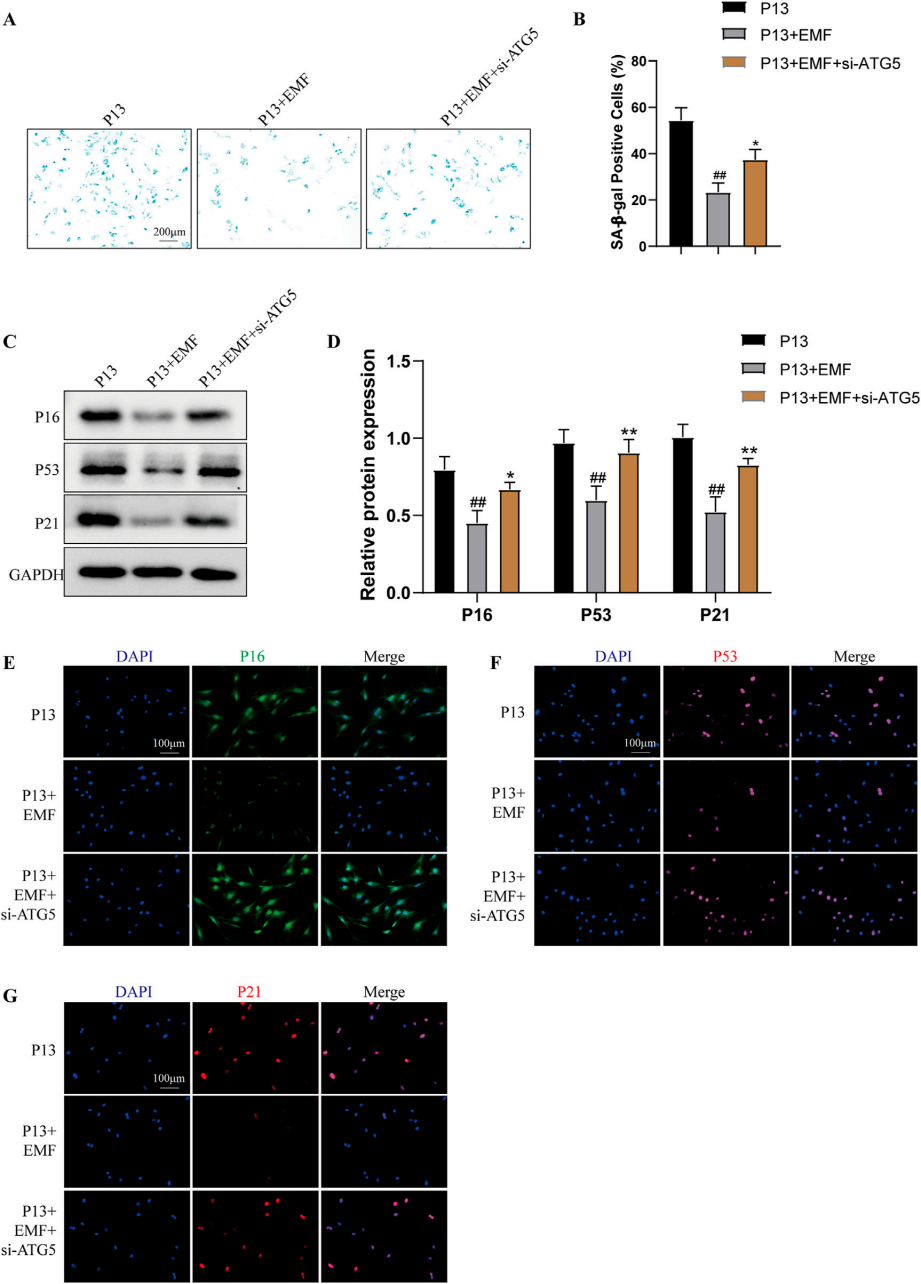
Knockdown of ATG5 blocks the rejuvenation effects of EMF in long-term passaged BMSCs P13 BMSCs were transfected with negative control siRNA (P13 group), negative control siRNA plus EMF (P13 + EMF group) or ATG5 siRNA plus EMF (P13 + EMF + siATG5 group) for 24 h. **(A)** SA-β-gal staining and **(B)** percentage of SA-β-gal-positive cells among three groups (n = 3). **(C)** Western blot and **(D)** Quantitative analysis of P16, P53, P21 among three groups. GAPDH served as the loading control (n = 3). **(E)** P16, **(F)** P53 and **(G)** P21 were further observed by Immunofluorescence among three groups. ^##^ *p* < 0.01 vs. P13 group; *p < 0.05 and **p < 0.01 vs. P13 + EMF group.

To illustrate whether EMF exposure inhibited BMSCs cell senescence by regulating autophagy, we used siRNA to knock down ATG5 as well as autophagy inhibitor chloroquine (CQ). As shown in Supplementary Figures S4, 24 h EMF exposure triggered the increased autophagy level in long passaged BMSCs (P13 BMSCs). P13 BMSCs showed decreased levels of SA-β-galactosidase activity after 24 h EMF treatment, while knockdown of ATG5 (Figures 7A, B) or using CQ (Supplementary Figures S5A, B) could antagonize this change. Cell senescence-associated proteins including P16, P53 and P21 were decreased in P13 BMSCs after 24 h EMF exposure. However, the Western blot assay (Figures 7C, D; Supplementary Figures S5C, D) and immunofluorescence staining (Figures 7E–G; Supplementary Figures S5E–G) revealed EMF lost its cell rejuvenation effects after knockdown of ATG5 or treatment of CQ in P13 BMSCs. Taken together, EMF delayed long passaged BMSCs senescence partly via promoting autophagy.

### 3.6 EMF preserved the therapeutic effect of long-term passaged BMSCs in bone regeneration

**FIGURE 8:**
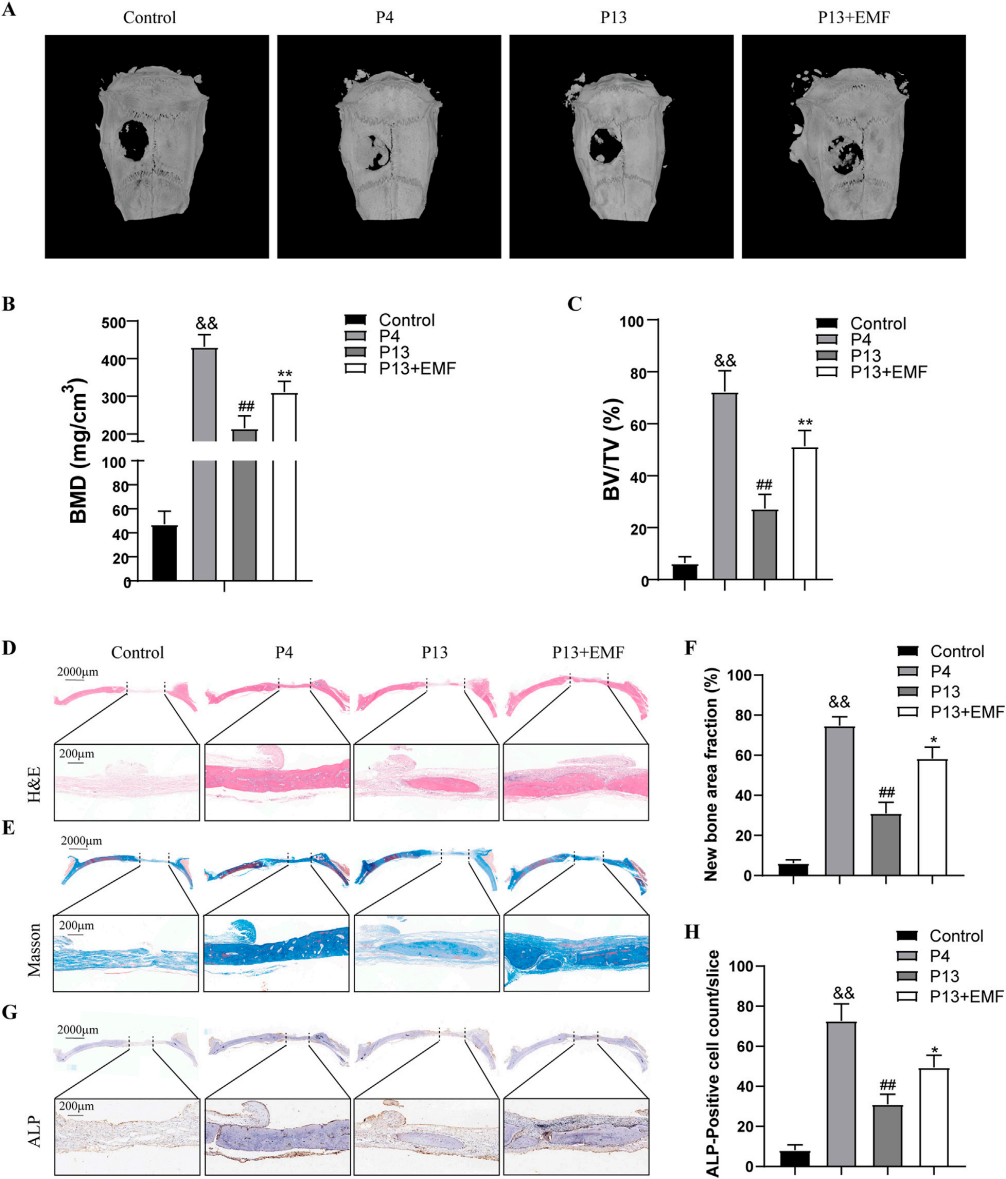
Restoration of the bone regeneration potential of aged BMSCs *in vivo* by EMF **(A)** Representative reconstructed micro-CT images of cranial defect in four groups 8 weeks after surgery. **(B)** BMD and **(C)** BV/TV quantification analysis among four groups (n = 3). **(D)** HE and **(E)** Masson’s trichrome staining of cranial defect area 8 weeks post implantation. The dotted line above indicated the boundary of the 6-mm cranial defect. **(F)** Quantitative analysis of new bone area fraction among four groups (n = 3). **(G)** ALP Immunohistochemistry staining of the cranial defect area 8 weeks after surgery. **(H)** Quantitative analysis of percentage of ALP-positive cells among four groups (n = 3). ^&&^ *p* < 0.01 vs. Control group; ^##^ *p* < 0.01 vs. P4 group; *p < 0.05 and **p < 0.01 vs. P13 group.

To determine the effects of EMF on the regenerative capacity of long-term passaged BMSCs *in vivo*, we created a rat critical-sized calvarial defect model. Acellular GelMA hydrogel or GelMA hydrogels mixed with P4, P13 BMSCs were transplanted into the calvarial defects of rat for 8 weeks. For P13 BMSCs transplant, rats were randomly selected to be treated with EMF (50Hz, 0.4mT, 4h/day) or not. Micro-CT imaging indicated that the restorative effects of P13 BMSCs was significantly declined compared with P4 BMSCs, while EMF exposure could effectively ameliorate this tendency manifested by higher values of BMD and BV/TV (Figures 8A–C). Moreover, HE and Masson’s trichrome staining were carried out to evaluate the new bone formation of each group. New bone area fraction comparisons of each group at 8 weeks revealed that EMF exposure significantly enhanced the bone regeneration of long passaged BMSCs *in vivo* (Figures 8D–F). We also performed the immunohistochemistry staining of ALP in four groups. P13 BMSCs exhibited decreased osteogenic capacity compared with P4 BMSCs *in vivo*. However, EMF exposure could partly reverse this change (Figures 8G, H).

## 4 Discussion

Stem cell senescence during *in vitro* expansion notably hinders the efficacy of cell therapy (Liao et al., 2019). Finding reliable strategies for stem cell rejuvenation remains a great challenge in cyto-therapy. In this study, we offered a novel approach based on EMF treatment to delay BMSCs senescence owing to long-term *in vitro* expansion. After EMF exposure, aged BMSCs exhibited enhanced self-renewal, osteogenic, chondrogenic and reduced adipogenic capacity. Meanwhile, EMF treatment prevented the stemness loss and senescent phenotypes aggravation of BMSCs during long-term expansion. Mechanistically, EMF treatment functioned via promoting the autophagy level in aged BMSCs. Importantly, based on the rat critical-sized calvarial defect model, we certified that EMF exposure largely preserved the therapeutic effect of long-term passaged BMSCs in bone regeneration *in vivo*. Taken together, this study highlights the promising therapeutic potential of EMF treatment in BMSCs rejuvenation during long-term *in vitro* expansion.

Since bone marrow had an extremely low proportion of BMSCs, *in vitro* expansion is necessary for BMCSs-based therapeutic purpose (Derubeis and Cancedda, 2004). The BMSCs senescence accompanied with the cell passaging *in vitro* poses a big problem in front of us. In our study, BMSCs of passage 13 (P13) showed decreased proliferation, multiple differentiation, stemness as well as increased senescence phenotypes compared to BMSCs of passage 4 (P4). These results are in accordance with previous study (Shuai et al., 2016; Yuan et al., 2019). Recently, numerous promising strategies had been developed to rejuvenate aged stem cells. However, either application restrictions or potential risks of them push us to find more avenues (Brunet et al., 2023). EMF intervention with different parameters had been authorized by US Food and Drug Administration (FDA) and extensively applied in tissue engineering (Aaron et al., 2004). More and more studies confirmed that low frequency EMF treatment could promote the self-renewal and differential capacity of different BMSCs (Ross et al., 2015). This suggests us that EMF with suitable parameter may serve as a candidate for BMSCs rejuvenation. Moreover, unlike some rejuvenation interventions have only transient benefits, our previous study indicated EMF exposure displayed a persistent effect on BMSCs without the risk of gene mutation (Tu et al., 2018). Therefore, in the current study, sinusoidal EMF (50 Hz, 0.4 mT) was used as the treatment for cell aging. Date proved EMF treatment effectively delay the BMSCs senescence during long-term *in vitro* culture.

Decline in autophagy activity is closely linked with stem cell exhaustion and senescence (Revuelta and Matheu, 2017; Ho et al., 2017; Mendelsohn and Larrick, 2016). Improving autophagy contributes to restoring the regenerative capacity of senescent stem cells (Bi, Wang, and Kuang, 2018). Reportedly, physiological autophagy inducer has prominent protective effects in cell aging and age-associated diseases (Madeo et al., 2018; Madeo et al., 2019). In In our study, reduced autophagy level and PI3K/AKT/mTOR pathway activation is observed in P13 BMSCs compared to P4 BMSCs. Application of autophagy agonist rapamycin or overexpression of ATG5 in P13 BMSCs significantly relieve the cell senescence. Meanwhile, when ATG5 was silenced using siRNA in P4 BMSCs, the senescence markers including P21, P16 and P53 were notably increased. These results suggest autophagy reduction is directly related to the BMSCs aging during long-term *in vitro* expansion. We further detected that EMF treatment could enhance the autophagy and PI3K/AKT/mTOR phosphorylation in P13 BMSCs. To elucidate whether EMF exposure functions via regulating autophagy, we next silenced ATG5 in P13 BMSCs or treated BMSCs with autophagy inhibitor chloroquine. It is then intriguing to see that EMF exposure lost its rejuvenation effects in P13 BMSCs.

To verify whether EMF exposure promotes cell therapy of long-term passaged BMSCs, we performed the *in vivo* investigation using a rat critical-sized calvarial defect model. Previous studies indicate that critical-sized defect model creates an environment where bone fracture results in nonunion without extra interventions (Harris et al., 2013). Moreover, calvarial defects over 5 mm in diameter could be regarded as a critical-sized defect in adult rat (Vajgel et al., 2014). Using this model in our study enable more accurate comparison among different grafts. In this study, declined bone regenerative capacity was observed in BMSCs after long-term *in vitro* expansion. However, EMF treatment could largely reverse this trend. Since *in vitro* expansion of BMSCs is an indispensable step for cell grafting. Our *in vivo* results suggest EMF may be used as an efficient tool for improving BMSCs transplantation efficiency after long-term *in vitro* culture.

To our knowledge, EMF with different frequencies or intensities exhibited diverse biological effects. It will be interesting to investigate the effects of EMF with other parameters on stem cell senescence in the future study. Furthermore, based on this study, the direct molecular target of EMF in rejuvenating senescent BMSCs during long-term expansion is unknown, further work is still essential in the future.

## 5 Conclusion

Above all, this study demonstrated that EMF treatment effectively delayed BMSCs senescence during long-term *in vitro* expansion by enhancing autophagy. The *in vivo* rat skull defect repair assay further proved that EMF treatment preserved the therapeutic potential of aged BMSCs during long-term *in vitro* culture. Taken together, our findings may offer a novel avenue for the BMSCs-based tissue engineering and provided insights into the biological effects of EMF on stem cell aging.

## Data Availability

The original contributions presented in the study are included in the article/Supplementary Material, further inquiries can be directed to the corresponding author.
