# Supplementary material for: EMF treatment delays mesenchymal stem cells senescence during long-term in vitro expansion by modulating autophagy
Source: Front Cell Dev Biol. 2024 Oct 7;12:1489774. doi: 10.3389/fcell.2024.1489774 (PMC11491334; doi:10.3389/fcell.2024.1489774)
Supplement: Supplementary file 1 [file Table1.DOCX]

**Supplementary figures**


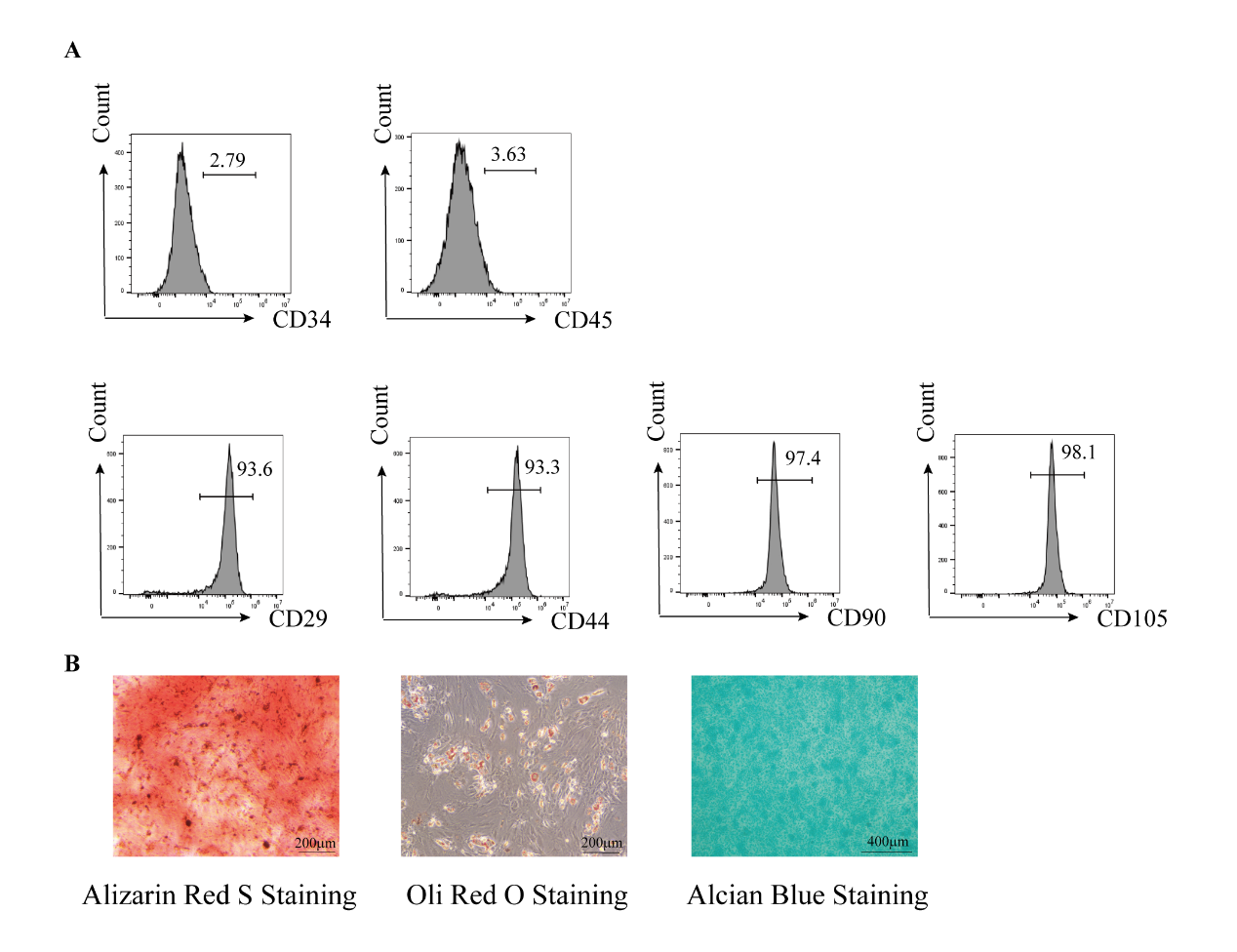


**Figure S1. Rat BMSCs identification**

Identification of rat BMSCs via detecting cell surface markers and MSC multipotential differentiation towards the osteogenic, adipogenic and chondrogenic lineages. (A) Cell surface markers detected using Flowcytometry. (B) Cells were cultured in osteogenic induction medium, adipogenic induction medium and chondrogenic induction medium respectively for 2 weeks. Afterwards, cells were stained with Arizarin Red S, Oil Red O and Alcian Blue.


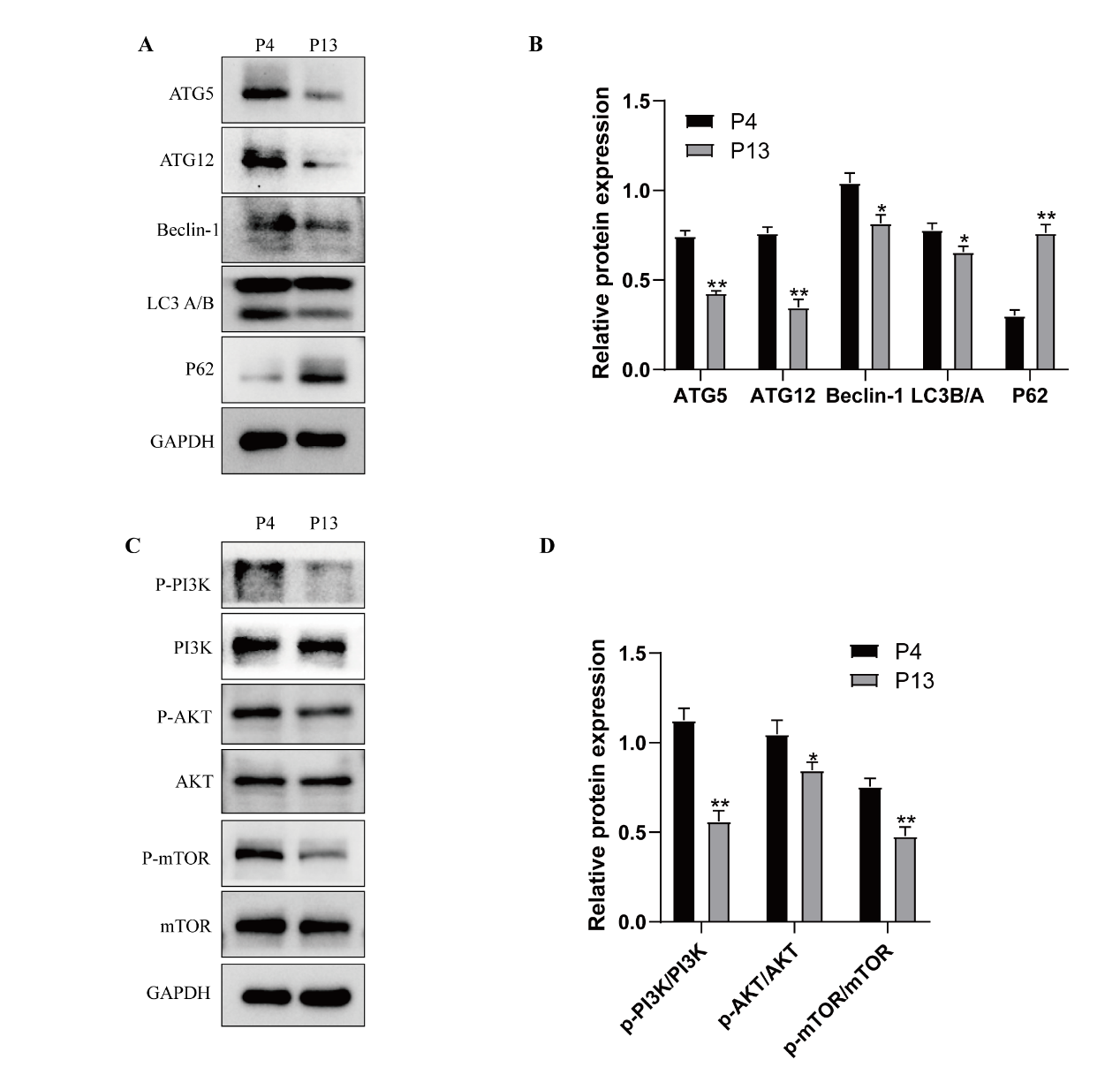


**Figure S2. Impaired autophagy is observed in BMSCs during long-term in vitro passage**

(A) Western blot and (B) Quantitative analysis of autophagy related proteins including ATG5, ATG12, Beclin-1, LC3B/A, P62 in P4 and P13 BMSCs. GAPDH was used as the internal controls (n=3). (C) Western blot and (D) Quantitative analysis of PI3K/AKT/mTOR signal pathways in P4 and P13 BMSCs. PI3K, AKT and mTOR were used as the internal controls (n=3). **p* < 0.05 and ***p* < 0.01 vs. P4 group.


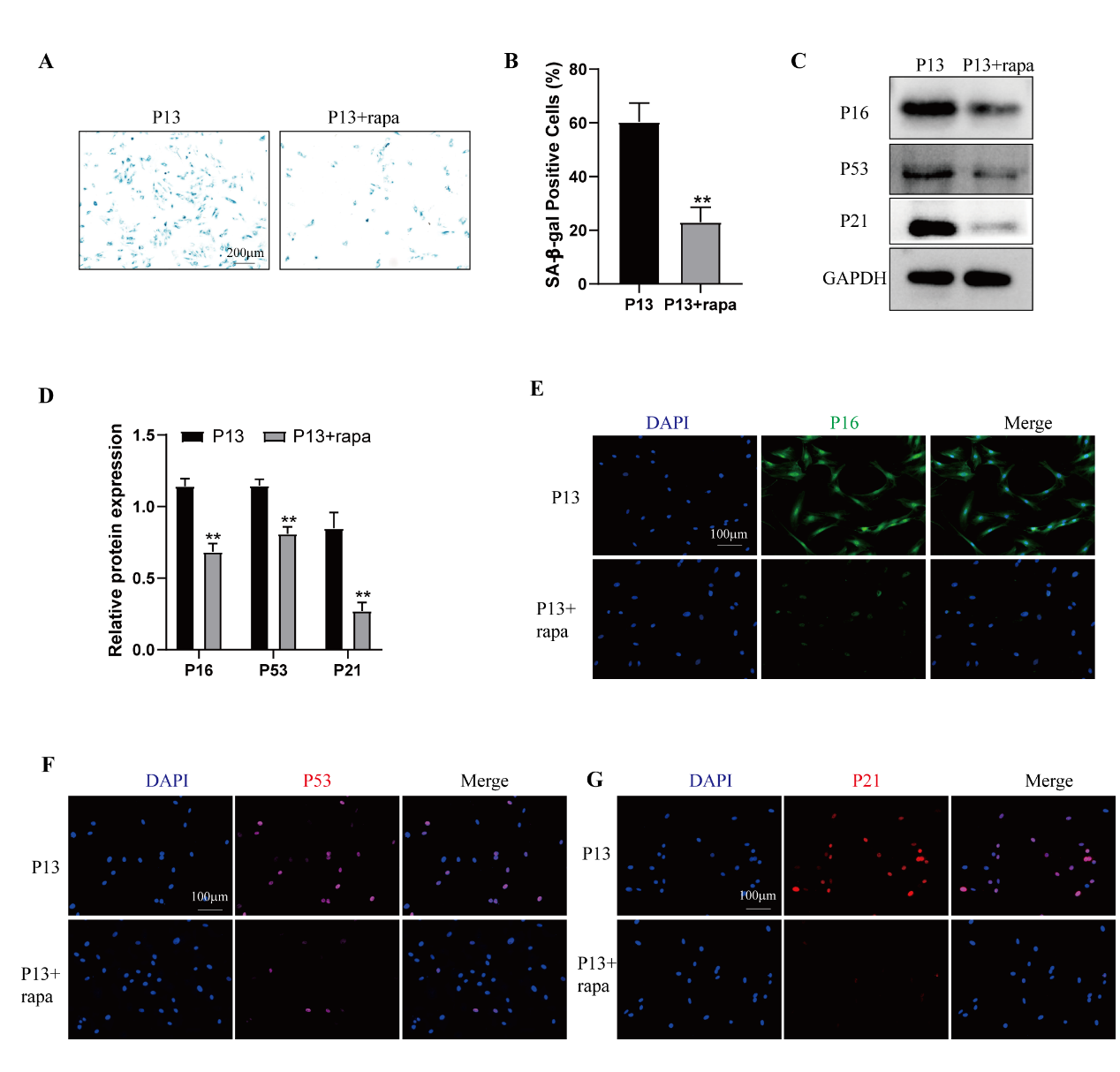


**Figure S3. Rapamycin ameliorates the cell senescence of long-term passaged BMSCs**

P13 BMSCs were treated with or without 100 nM rapamycin for 24h. (A) SA-β-gal staining and (B) percentage of SA-β-gal-positive cells of P13 and rapamycin-treated P13 BMSCs. (n=3). (C) Western blot and (D) Quantitative analysis of senescence makers including P16, P53, P21 in P13 and rapamycin-treated P13 BMSCs. GAPDH was employed as the internal control (n=3). (E) P16, (F) P53 and (G) P21 were further observed by Immunofluorescence among two groups. ***p* < 0.01 vs. P13 group.


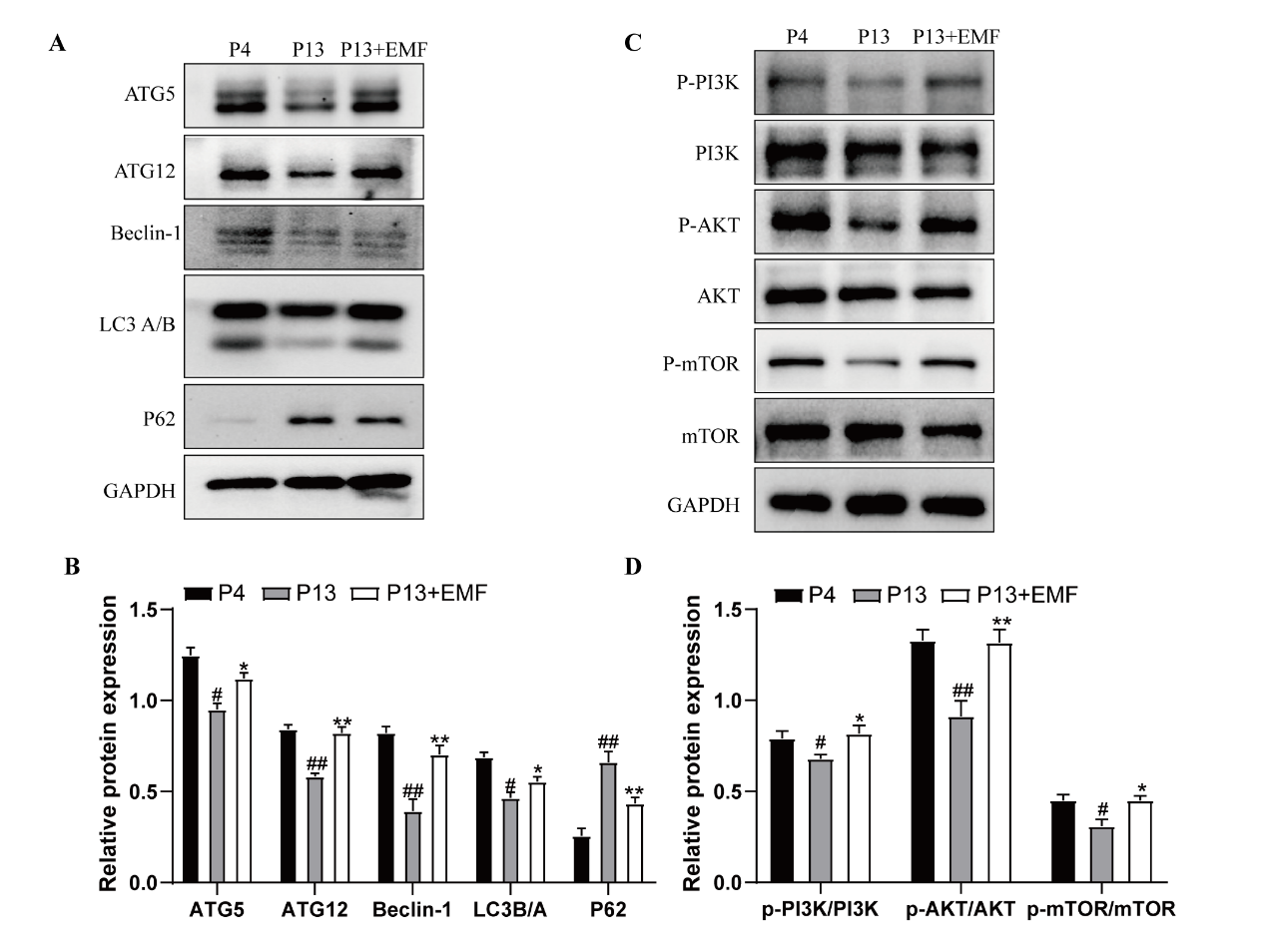


**Figure S4. EMF exposure enhanced the autophagy in long-term passaged BMSCs**

(A) Western blot and (B) Quantitative analysis of autophagy related proteins including ATG5, ATG12, Beclin-1, LC3B/A, P62 in P4, P13 and 24h EMF-stimulated P13 BMSCs. GAPDH served as the internal controls (n=3). (C) Western blot and (D) Quantitative analysis of PI3K/AKT/mTOR signal pathways in P4, P13 and EMF-stimulated P13 BMSCs. PI3K, AKT and mTOR served as the loading controls (n=3). ^#^*p* < 0.05 and ^##^*p* < 0.01 vs. P4 group; **p* < 0.05 and ***p* < 0.01 vs. P13 group.


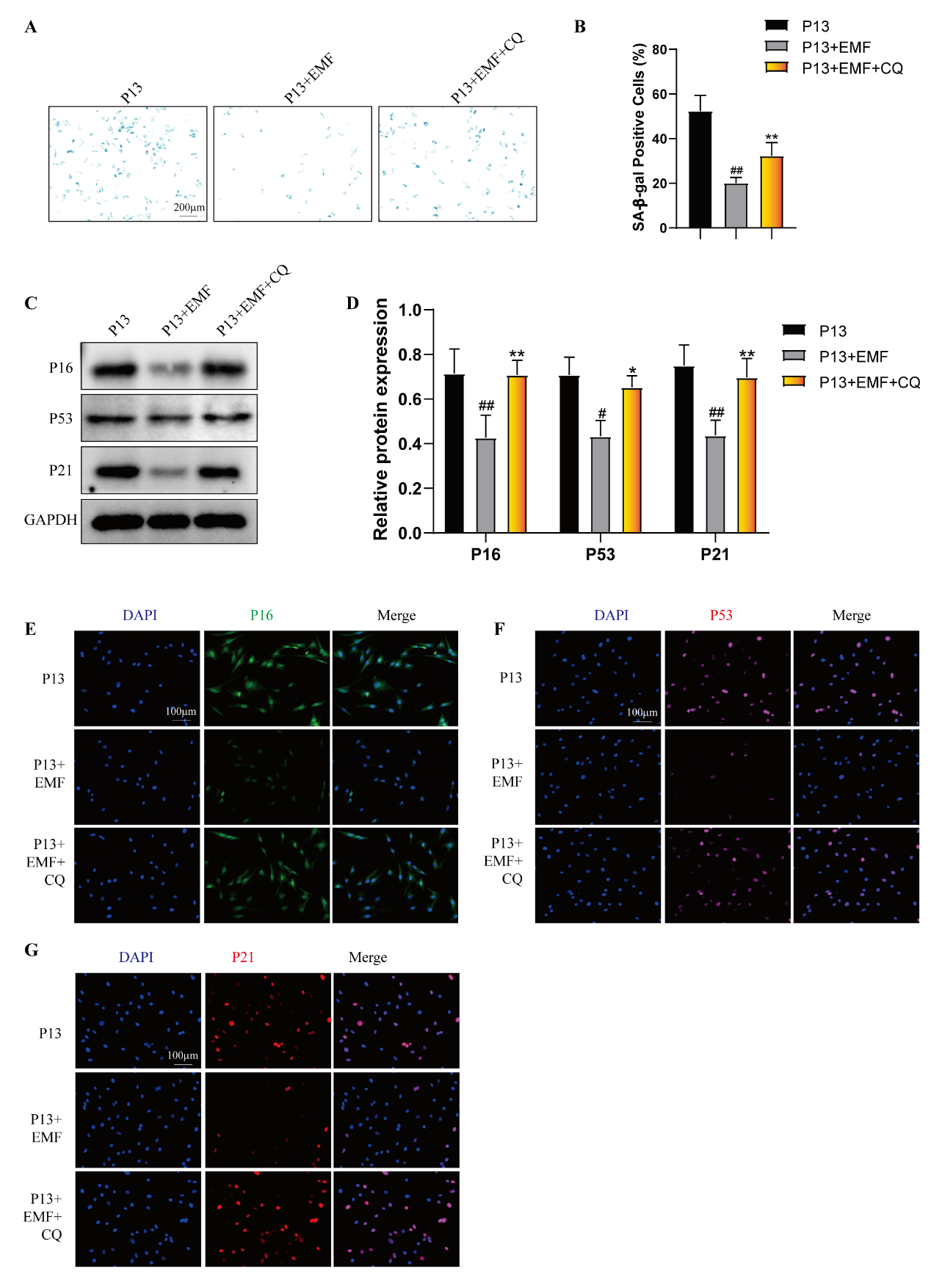


**Figure S5. Chloroquine antagonizes the rejuvenation effects of EMF in long-term passaged BMSCs**

P13 BMSCs were treated with 50μM CQ plus EMF (P13+EMF+CQ group), equal volumes of PBS (P13 group) or equal volumes of PBS plus EMF (P13+EMF group) for 24h. (A) SA-β-gal staining and (B) percentage of SA-β-gal-positive cells among three groups (n=3). (C) Western blot and (D) Quantitative analysis of P16, P53, P21 among three groups. GAPDH was used as the internal control (n=3). (E) P16, (F) P53 and (G) P21 were further observed by Immunofluorescence among three groups. ^#^*p* < 0.05 and ^##^*p* < 0.01 vs. P13 group; **p* < 0.05 and ***p* < 0.01 vs. P13+EMF group.


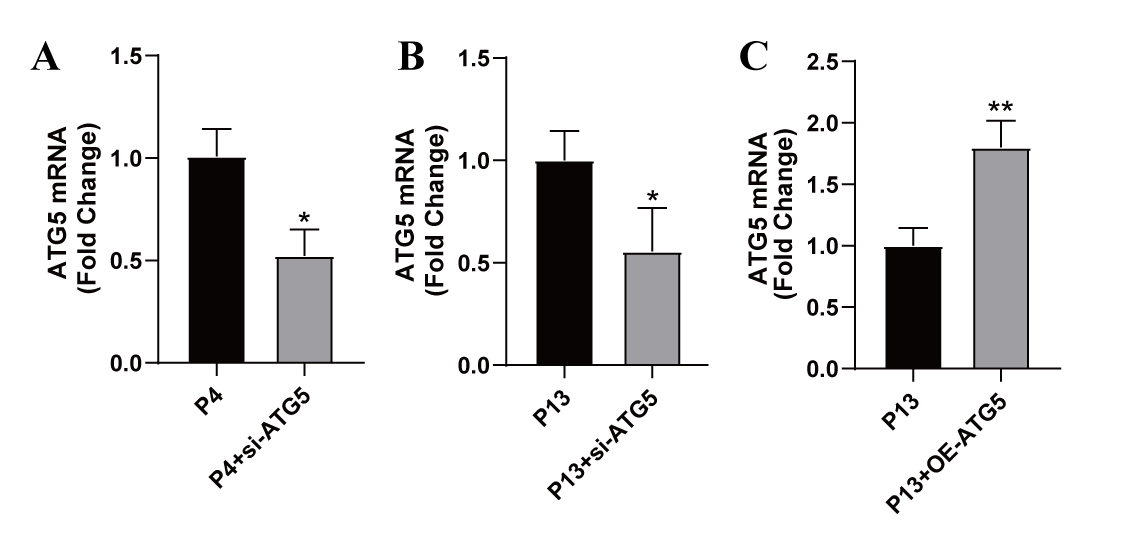


**Figure S6. Knockdown and over-expression of ATG5 in P4 or P13 BMSCs were verified by qPCR**

(A) P4 BMSCs were transfected with negative control siRNA (P4 group) or ATG5 siRNA (P4+siATG5 group) for 24h. ATG5 mRNA levels of two group were analyzed by qPCR. (B) P13 BMSCs were transfected with negative control siRNA (P13 group) or ATG5 siRNA (P13+siATG5 group) for 24h. ATG5 mRNA levels of two group were analyzed by qPCR. (C) P13 BMSCs were transfected with empty vectors (P13 group) or Ad-ATG5 adenoviruses vectors (P13 + OE-ATG5 group) for 24h. ATG5 mRNA levels of two group were analyzed by qPCR. **p* < 0.05 and ***p* < 0.01.
